# Longitudinal changes in desired body weight compared to changes in body weight: evidence of adaptation to weight gain?

**DOI:** 10.1186/s40608-016-0120-6

**Published:** 2016-09-21

**Authors:** Nils Abel Aars, Bjarne K. Jacobsen

**Affiliations:** Department of Community Medicine, UiT – The Arctic University of Norway, Tromsø, Norway

**Keywords:** Ideal body weight, Longitudinal study, Body weight change

## Abstract

**Background:**

Overweight individuals desire a lower weight than they actually have. Little is known on the extent to which this discrepancy is reduced over time due to adaptation or resignation. The aim of this study is to describe cross-sectional relationships and longitudinal changes in desired body weight and differences between actual and desired body weight according to gender, age and category of body mass index in a large, adult cohort in Tromsø, Norway.

**Methods:**

Cross-sectional analyses of 8960 men and 9992 women aged 25–69 participating in Tromsø 4 (during 1994–1995), and longitudinal analyses of 3210 men and 3689 women participating in both Tromsø 4 (during 1994–1995) and Tromsø 6 (during 2007–2008). Simple descriptive statistics and linear regression was used to describe actual and desired weight, the difference between them, and how gender and age are related to the changes in actual and desired weight over this 13-year period.

**Results:**

The difference between actual and desired body weight was largest for the obese and higher among the overweight than the normal weight for both genders. While normal weight men were quite satisfied with their body weight, normal weight women were not. Actual weight increased more than desired weight for all age groups and both genders except the oldest women. The difference between change in actual body weight and change in desired body weight during the 13-year follow-up was significantly larger among men (2.0 kg) than women (1.5 kg) (*p* < 0.001), and larger among young than older adults (*p* < 0.001). Adjusting for level of education had no impact on this relationship. Furthermore, the association between age and the difference between change in actual body weight during the 13 years and change in desired body weight in the same period did not differ between men and women and, in gender specific analyses, between subjects with normal weight and those who were overweight or obese at start of follow-up.

**Conclusion:**

Older people adapt more to weight gain than younger age groups, with clear gender differences. Further studies of longitudinal changes in desired weight are warranted.

**Electronic supplementary material:**

The online version of this article (doi:10.1186/s40608-016-0120-6) contains supplementary material, which is available to authorized users.

## Background

In Western countries, dissatisfaction with body weight or body shape is prevalent [1–3] and attempts to reduce weight, often in vain [4], are endemic [5, 6]. Weight dissatisfaction has been found to vary with age, gender, level of education and body mass index (BMI) [7, 8], which is important as a small difference between actual- and desired body weight has been associated with improved health status [9–11]. Blake et al. found that overweight men who were satisfied with their weight had lower blood pressure and serum total cholesterol than those who were dissatisfied. Among obese men, significant differences were also found between those satisfied and those dissatisfied with their weight. These differences seem to be weaker in women [9]. According to Muennig et. al., the percentage of desired weight loss was a stronger predictor of past unhealthy days than BMI, implying that the difference between actual- and desired bodyweight plays a greater role in generating disease than adiposity itself [11]. Therefore, it is important to obtain a deeper understanding of how the difference between actual- and desired body weight varies according to gender, age or category of BMI [12, 13]. In a longitudinal perspective, it is also of interest to investigate whether actual and desired body weight, and the difference between them, remain stable over time. Longitudinal changes in BMI and body weight has been subject for several studies [14–18], but little is known about longitudinal changes in desired body weight in relation to changes in actual body weight [19]. For policy makers and public health officials, information about attitudes towards weight gain is important, especially in light of studies suggesting that obesity is “socially contagious” and that higher body weights have become more acceptable over time [10, 20, 21]. From a clinical perspective, the issue of weight loss expectations may benefit from research on longitudinal changes in desired weight, especially with regard to potential adaptation to weight gain in BMI subgroups.

The focus of the current paper is therefore on peoples’ desired weights. The aim is to describe in a longitudinal study how desired body weight, and the difference between actual and desired body weight, differs across gender, age and BMI categories using actual, measured body weight and self-reported desired body weight. To our knowledge, this is the first population-based longitudinal analysis providing evidence concerning the extent to which discrepancies between actual- and desired body weight changes over time. Furthermore, the results across gender and age groups provide important insights into who are most likely to adapt to (and accept) their increased actual weight.

## Methods

The Tromsø Study is a large health survey, based on the population in the city of Tromsø, Norway [22]. The Tromsø study was conducted for the first time in 1974 and has been repeated five times since then, with a seventh survey currently in progress. The present longitudinal analyses are based on the fourth and sixth Tromsø Study (Tromsø 4 and Tromsø 6), conducted in 1994–1995 and 2007–2008, respectively. Data from the fifth Tromsø Study (Tromsø 5) was available, but was not included in the longitudinal analyses since this would give a smaller sample size for the longitudinal analyses (2739 men and women compared to 6899 subjects).

The design of the Tromsø Study has been described in detail elsewhere [18, 22, 23] and only the most relevant information is given here. In the fourth Tromsø Study, the entire population of Tromsø born before 1970 was invited, and the participation rate was 72 %, 27 158 attenders out of the 37 558 invited. In 2001, the fifth Tromsø Study was conducted, inviting all 30-, 40-, 45-, 60- and 75-year olds in the county of Tromsø, as well as all participants from a second visit in Tromsø 4 [22]. In total 10 353 men and women were invited, with 8130 participating (participation rate of 79 %). The sixth Tromsø study was conducted in 2007–2008 and had 12 984 participants, out of 19 762 invited, giving a participation rate of 66 %. The invited population was partly based on attendees to Tromsø 4 and partly of complete birth cohorts and random samples from the age groups 30–87 [23].

At all three surveys the clinical examination included measurements of height and weight with minimal clothing. Height was measured to the nearest 1 cm in Tromsø 4 and to the nearest 0.1 cm in Tromsø 5 and Tromsø 6. Weight was measured to the nearest 500 g in Tromsø 4, and to the nearest 100 g in Tromsø 5 and Tromsø 6 [18]. The actual body mass index (BMI) was computed as weight (kg)/height (meter) ^2^ (kg/m^2^). The participants were asked to complete a self-administered questionnaire that included the question “*What weight would you be satisfied with (your “ideal” weight)?”*. In Tromsø 4, this question was included only in the questionnaire issued to participants below 70 years of age. The cross-sectional analyses from Tromsø 4 and the longitudinal analyses were therefore restricted to those between the age of 25 and 69 at the time of Tromsø 4. A total of 33 446 men and women aged 25–69 were invited, and data on desired weight was available for 57 % of them.

Information about current pregnancy was obtained by questionnaires and interview during height and weight measurements. Information concerning years of education was available from Tromsø 4, with five possible responses; 1. Elementary school, 2. Vocational high school, 3. High school, 4. College/university <4 years, and 5. College/university 4 years or more.

For the present analyses, we selected men and non-pregnant women who consented to participate in research, who had a valid measurement of body weight and had answered the question of what body weight they would be satisfied with. In total, 8960 men and 9992 women participating in Tromsø 4 were included. The corresponding numbers from Tromsø 5 was 2940 men and 3750 women, while 5012 men and 5538 women were included from Tromsø 6. The difference between actual- and desired body weight was computed by subtracting desired body weight from actual bodyweight.

The longitudinal analyses included 3210 men and 3689 women that participated in both Tromsø 4 and Tromsø 6. We computed the difference in actual body weight between Tromsø 4 and 6 (actual difference in weight), the difference in desired body weight between Tromsø 4 and 6 (difference in desired weight), as well as the difference between the two differences (discrepancy in difference). The latter reflects the extent that the subject has adapted to the (in most situations) increase in body weight.

In our sample, 5756 men and 6295 women in Tromsø 4 answered the question of desired weight in Tromsø 4, but did not provide an answer to the question of desired weight in Tromsø 6 as they were e.g., not invited to the survey, had moved or died. For men, after adjustment for age, desired weight differed slightly (200 g), but statistically significantly (*p* < 0.001), between those that answered the question of desired weight in Tromsø 4 only and those that had answered the question in both Tromsø 4 and Tromsø 6. For women there was no age-adjusted difference (*p* = 0.7).

All results were presented gender-specific according to 5-year age groups (age in 1994) and three categories of BMI (normal weight: BMI <25 kg/m^2^, overweight: BMI 25–25.9 kg/m^2^ and obesity: BMI ≥30 kg/m^2^). As there were few subjects (2.2 % in Tromsø 4) who were underweight (BMI <18.5 kg/m^2^) [18], we merged these subjects with the group of normal weight. In the analyses, we present cross-sectional data from Tromsø 4 and longitudinal data from Tromsø 4 to Tromsø 6. Cross-sectional results from Tromsø 5 and Tromsø 6 were also analyzed, but are not included in the present paper. These results are accessible to readers through the Additional file 1.

All statistical analyses were performed using SPSS V.22 for Windows and included descriptive statistics and linear regression. Results were considered significant if *p* < 0.05. The Tromsø Study was approved by the Regional Committee for Research Ethics.

## Results

Tables 1 and 2 give the cross-sectional distribution of actual and desired bodyweight, as well as the difference between the two, among men and women in Tromsø 4 according to 5-year age groups and three categories of BMI (normal weight, overweight and obesity).

**Table 1:** Cross-sectional analysis of measured and desired weight, and the difference between them, among men in Tromsø 4 (1994–1995)^a^

	Total				BMI <25				BMI 25-29.99				BMI ≥30			
Age group	*n*	Weight	Desired weight	Difference^b^ (CI)	*n*	Weight	Desired weight	Difference^b^ (CI)	*n*	Weight	Desired weight	Difference^b^ (CI)	*n*	Weight	Desired weight	Difference^b^ (CI)
25–29	1183	80.0	76.9	3.1 (2.8, 3.6)	670	73.6	74.3	−0.6 (-0.9, -0.4)	435	85.6	79.5	6.3 (5.9, 6.7)	78	102.8	85.2	17.6 (16.1, 19.4)
30–34	1190	80.4	76.5	3.9 (3.5, 4.3)	641	73.7	73.5	0.1 (-0.2, 0.4)	458	85.6	79.1	6.5 (6.1, 6.9)	91	101.9	84.6	17.3 (15.5, 19.1)
35–39	1268	81.8	77.2	4.7 (4.3, 5.0)	612	74.6	73.8	0.7 (0.5, 1.2)	564	86.1	79.2	6.8 (6.4, 7.2)	92	103.9	86.8	17.2 (15.9, 19.0)
40–44	1269	81.2	76.6	4.6 (4.3, 4.9)	575	73.3	72.7	0.5 (0.1, 0.8)	574	84.7	78.5	6.2 (5.8, 6.5)	120	102.0	85.4	16.5 (15.2, 18.0)
45–49	1219	83.1	77.6	5.5 (5.0, 5.9)	423	72.5	72.3	0.2 (-0.1, 0.6)	635	85.2	79.1	6.1 (5.7, 6.5)	161	102.1	85.6	16.5 (15.3, 17.8)
50–54	1002	83.0	77.6	5.4 (4.9, 5.7)	344	72.7	72.0	0.7 (0.3, 1.1)	524	85.3	79.3	6.0 (5,7, 6.5)	134	100.1	85.4	14.7 (13.5, 16.1)
55–59	724	81.4	76.8	4.6 (4.2, 5.1)	270	71.8	71.7	0.1 (-0.3, 0.5)	373	84.4	78.7	5.7 (5.2, 6.1)	81	99.8	85.2	14.6 (12.9, 15.8)
60–64	598	80.8	75.9	5.0 (4.5, 5.7)	201	70.5	70.5	0.0 (-0.6, 0.4)	328	83.0	77.6	5.4 (5.1, 5.7)	69	100.6	83.4	17.3 (15.4, 19.2)
65–69	507	78.7	74.7	4.0 (3.4, 4.6)	204	69.2	70.2	−1.0 (-1.6, -0.5)	244	82.3	77.0	5.3 (4.7, 5.8)	59	96.8	80.7	16.1 (14.2, 18.0)
Total	8960	81.3	76.8	4.5 (4.3, 4.6)	3940	73.0	72.9	0.1 (0.0, 0.3)	4135	84.9	78.8	6.1 (6.0, 6.2)	885	101.3	85.0	16.3 (15.8, 17.0)

^a^:Results for all 8960 men and according to three categories of BMI (normal weight, overweight and obese)

^b^Difference: Difference between actual, measured weight and self-reported desired weight, with 95 % confidence intervals

**Table 2:** Cross-sectional analysis of measured and desired weight, and the difference between them, among women in Tromsø 4 (1994–1995)^a^

	Total				BMI < 25				BMI 25-29.99				BMI ≥ 30			
Age group	*n*	Weight	Desired weight	Difference (CI)^b^	*n*	Weight	Desired weight	Difference (CI)^b^	*n*	Weight	Desired weight	Difference (CI)^b^	*n*	Weight	Desired weight	Difference (CI)^b^
25–29	1391	64.2	58.7	5.6 (5.3, 5.9)	1029	59.8	56.8	3.0 (2.8, 3.2)	283	73.0	62.7	10.3 (9.8, 10.7)	79	90.4	68.3	22.1 (20.2, 23.8)
30–34	1434	64.3	58.9	5.4 (5.1, 5.8)	1056	59.9	57.0	2.9 (2.7, 3.2)	302	73.1	62.8	10.3 (9.9, 10.8)	76	90.2	69.4	20.8 (18.7, 22.9)
35–39	1468	65.3	59.4	5.8 (5.5, 6.2)	1053	60.7	57.6	3.1 (2.9, 3.3)	323	73.0	63.1	9.9 (9.4, 10.3)	92	90.7	67.8	22.9 (20.9, 24.9)
40–44	1403	65.3	59.4	6.0 (5.7, 6.4)	944	60.8	57.8	3.0 (2.8, 3.2)	352	72.4	63.0	9.3 (8.9, 9.8)	107	89.2	67.2	22.0 (20.0, 23.5)
45–49	1354	67.5	60.9	6.6 (6.2, 6.8)	764	60.9	58.0	2.8 (2.6, 3.0)	447	72.3	63.5	8.8 (8.5, 9.1)	143	87.8	68.4	19.4 (18.1, 20.9)
50–54	1017	68.8	61.7	7.1 (6.6, 7.5)	514	60.8	58.3	2.5 (2.2, 2.8)	352	72.2	63.9	8.3 (7.8, 8.7)	151	88.5	68.2	20.3 (18.9, 22.0)
55–59	730	68.9	62.0	6.9 (6.4, 7.7)	313	60.3	58.4	1.9 (1.5, 2.2)	301	71.4	63.6	7.9 (7.4, 8.3)	116	85.6	67.4	18.2 (16.6, 19.7)
60–64	612	67.5	61.6	5.9 (5.3, 6.5)	251	58.7	57.6	1.1 (0.8, 1.5)	267	70.0	63.2	6.7 (6.3, 7.3)	94	83.8	67.3	16.4 (15.3, 18.1)
65–69	583	68.6	62.5	6.2 (5.5, 6.8)	236	58.4	57.7	0.8 (0.1, 1.4)	224	70.5	64.2	6.4 (5.9, 6.8)	123	84.7	68.6	16.2 (14.8, 17.5)
Total	9992	66.3	60.2	6.1 (6.0, 6.2)	6160	60.3	57.6	2.7 (2.6, 2.8)	2851	72.1	63.3	8.7 (8.6, 8.9)	981	87.7	68.1	19.6 (19.0, 20.2)

^a^:Results for all 9992 women and according to three categories of BMI (normal weight, overweight and obese)

^b^Difference (CI): Difference between actual, measured weight and self-reported desired weight, with 95 % confidence intervals

Among all men, the difference between actual- and desired body weight was 4.5 kg (Table 1). For those with normal bodyweight, that is a BMI of less than 25 kg/m^2^, the difference was negligible (0.1 kg). In overweight men (BMI between 25 kg/m^2^ and 29.9 kg/m^2^) the difference was 6.1 kg. For obese men there was a marked difference, 16.3 kg. There was in all men a statistically significant (*p* < 0.001), but not very strong, association between age and difference between actual- and desired bodyweight, with a weak not statistically significant (*p* = 0.6) positive relationship for the normal weight and a negative relationship for those who were overweight or obese (*p* < 0.001) (p-value for interaction < 0.001). BMI category was a strong predictor of difference between actual- and desired body weight (*p* < 0.001).

Among all women there was a difference of 6.1 kg between actual- and desired body weight (Table 2), in normal weight women it was 2.7 kg, and in overweight 8.7 kg. In the group of obese women (BMI ≥30 kg/m^2^), the difference was 19.6 kg. Both age-group and category of BMI were found to be significant predictors of difference between actual- and desired bodyweight (*p* < 0.001). For all three BMI categories, the relationship with age was linear and inverse (*p* < 0.001), but the strength of the relationship differed by BMI category (*p* < 0.001).

There was no significant interaction with gender (*p* = 0.6), i.e., the overall relationship between age and difference between actual and desired weight in Tromsø 4 did not differ between men and women. The cross-sectional results for Tromsø 5 and Tromsø 6 were similar to those for Tromsø 4 (see Additional file 1: Tables S1–S4).

The longitudinal results displayed in Table 3 are based on 3210 men and 3689 women who participated in both Tromsø 4 and Tromsø 6. The table shows, for each gender and age group, first the difference in actual body weight between Tromsø 4 and Tromsø 6 (“Actual difference”, left column), then the difference in desired body weight between Tromsø 4 and Tromsø 6 (“Difference in desired weight”, middle column), and, finally, the difference between the two measures (“Discrepancy in difference”, right column).

**Table 3:** Longitudinal analyses of 13-year change in weight (kg), desired weight (kg) and difference between the two (kg), from Tromsø 4 to Tromsø 6

	Men				Women			
Age groups	n	Actual difference (CI)^a^	Difference in desired weight (CI)^b^	Discrepancy in difference (CI)^c^	n	Actual difference (CI)^a^	Difference in desired weight (CI)^b^	Discrepancy in difference (CI)^c^
25–29	345	7.7 (6.8, 8.5)	3.8 (3.4, 4.4)	3.9 (3.3, 4.8)	460	8.1 (7.4, 9.0)	5.2 (4.9, 5.7)	2.8 (2.3, 3.5)
30–34	243	7.3 (6.5, 8.0)	4.6 (4.0, 5.2)	2.8 (1.7, 3.5)	336	6.5 (5.8, 7.3)	4.3 (3.8, 4.7)	2.2 (1.7, 2.9)
35–39	301	6.2 (5.5, 6.9)	3.7 (3.1, 4.4)	2.5 (1.9, 3.3)	355	6.4 (5.7, 7.1)	4.3 (3.9, 4.7)	2.1 (1.5, 2.7)
40–44	321	5.4 (4.6, 6.4)	3.0 (2.5, 3.4)	2.4 (1.5, 3.1)	378	5.4 (4.9, 6.1)	3.6 (3.1, 4.0)	1.9 (1.3, 2.5)
45–49	593	3.9 (3.4, 4.4)	1.9 (1.6, 2.3)	2.0 (1.5, 2.5)	677	4.8 (4.3, 5.2)	3.3 (3.1, 3.6)	1.5 (1.0, 1.9)
50–54	638	3.0 (2.5, 3.4)	1.3 (0.9, 1.6)	1.6 (1.2, 2.1)	628	4.5 (3.9, 5.0)	3.0 (2.7, 3.2)	1.5 (1.1, 2.0)
55–59	394	2.1 (1.5, 2.8)	1.1 (0.7, 1.6)	1.0 (0.4, 1.5)	399	2.3 (1.8, 2.9)	2.2 (1.9, 2.6)	0.1 (−0.6, 0.7)
60–64	253	0.4 (-0.6, 1.1)	0.1 (-0.4, 0.6)	0.3 (-0.5, 0.8)	280	1.7 (0.7, 2.5)	1.2 (0.7, 1.6)	0.5 (-0.3, 1.3)
65–69	122	0.9 (-0.1, 1.9)	0.1 (-0.8, 0.8)	0.9 (-0.1, 1.8)	176	−0.3 (-1.7, 0.7)	0.4 (-0.4, 1.1)	−0.7 (-1.7, 0.4)
Total	3210	4.2 (3.9, 4.4)	2.2 (2.0, 2.3)	2.0 (1.7, 2.2)	3689	4.8 (4.5, 5.0)	3.3 (3.2, 3.4)	1.5 (1.3, 1.7)

^a^Actual difference (CI): Measured body weight in Tromsø 6 (2007–2008) minus measured weight in Tromsø 4 (1994–1995), with 95 % confidence intervals

^b^Difference in desired weight (CI): Desired weight in Tromsø 6 (2007–2008) minus desired weight in Tromsø 4 (1994–1995), with 95 % confidence intervals

^c^Discrepancy in difference (CI): Actual difference minus Difference in desired weight, with 95 % confidence intervals

In men, the mean change in actual body weight during the 13 years between the two surveys was 4.2 kg, and in women 4.8 kg. The largest differences were found in the youngest age group, 7.7 kg in men and 8.1 kg in women, respectively, and there was a statistically significant inverse linear relationship (*p* < 0.001) between age and the change in body weight. Similar results were found for the change in desired body weight (mean change in men and women was 2.2 kg and 3.3 kg, in men and women, respectively). The difference between the two (the extent to which change in actual weight exceeds change in desired weight) was significantly different between genders, 2.0 kg in men and 1.5 kg in women (*p* < 0.001). Thus, the change in actual weight was larger than change in desired body weight, and women adapted slightly more to weight gain than men did. The difference between the change in actual weight and the change in desired weight was clearly inversely related to age; the difference was not statistically significant in men aged 60 and above and women aged 55 and above. The linear relationship between age and the discrepancy in difference did not differ between men and women (*p* = 0.5). Adjustments for level of education had only marginal influence on the extent of adaptation to weight gain, i.e., the linear relationship between age and the discrepancy in difference. By dividing changes in desired weight by changes in actual weight, we found that men adjusted to around 52 % of their weight gain (2.2 kg/4.2 kg) and women adjusted to around 69 % of their weight gain (3.3 kg/4.8 kg).

Tables 4 and 5 are extensions of Table 3, and provide the longitudinal changes in actual- and desired body weight according to three different categories of BMI. For men (Table 4) there was a significant pattern of declining difference with age between the actual and desired change in weight for normal weight and overweight men (*p* < 0.001). For obese men, a clear pattern was not apparent (*p*-value for linear trend = 0.2), and in all but one age group, the change in weight was actually not statistically significantly different from the change in desired weight. The difference between the actual and desired change in weight was not significantly associated with category of BMI (*p* = 0.2), and no statistical interaction by BMI category was found when assessing the relationship between age and the difference between the actual and desired change in weight (*p* = 0.7).

**Table 4:** Longitudinal analyses of 13-year change in weight (kg), desired weight (kg) and difference between the two (kg), among men according to BMI category in Tromsø 4

	BMI <25				BMI 25-29.99				BMI ≥30			
	n	Actual difference^a^	Difference in desired weight^b^	Discrepancy in difference (CI)^c^	n	Actual difference^a^	Difference in desired weight^b^	Discrepancy in difference (CI)^c^	n	Actual difference^a^	Difference in desired weight^b^	Discrepancy in difference (CI)^c^
25–29	197	7.9	4.0	3.9 (3.1, 4.5)	121	7.9	3.7	4.3 (3.0, 5.3)	27	5.8	3.1	2.6 (-2.3, 7.0)
30–34	125	7.5	4.4	3.1 (2.3, 3.8)	102	7.3	4.4	2.9 (1.6, 4.3)	16	5.6	6.2	−0.6 (-8.4, 5.4)
35–39	133	5.9	3.4	2.4 (1.4, 3.1)	146	6.3	3.5	2.7 (1.8, 3.8)	22	8.2	6.7	1.5 (-1.8, 4.5)
40–44	133	5.2	3.0	2.2 (1.4, 3.0)	152	5.9	3.2	2.7 (1.8, 3.9)	36	4.5	2.4	2.1 (-1.2, 5.1)
45–49	182	4.4	2.1	2.3 (1.7, 3.1)	332	3.7	1.8	2.0 (1.5, 2.8)	79	3.1	1.9	1.2 (-0.7, 3.1)
50–54	212	2.6	1.4	1.2 (0.3, 2.0)	333	3.2	1.4	1.8 (1.4, 2.4)	93	3.1	1.2	1.9 (0.2, 3.6)
55–59	139	2.3	1.3	1.0 (0.3, 1.8)	215	1.9	0.9	1.0 (0.4, 1.8)	40	2.8	1.6	1.2 (-1.4, 4.0)
60–64	83	0.9	0.0	0.9 (0.1, 1.8)	144	0.4	0.0	0.4 (-0.5, 1.3)	26	−1.4	0.9	−2.3 (-4.7, -0.1)
65–69	45	0.8	0.5	0.2 (-1.2, 1.9)	63	1.1	−0.5	1.6 (0.0, 3.5)	14	0.7	1.2	−0.5 (-4.2, 3.4)
Total	1249	4.6	2.5	2.1 (1.9, 2.4)	1608	4.0	1.9	2.0 (1.7, 2.3)	353	3.4	2.2	1.2 (0.4, 2.1)

^a^Actual difference: Measured body weight in Tromsø 6 (2007–2008) minus measured weight in Tromsø 4 (1994–1995), with 95 % confidence intervals

^b^Difference in desired weight: Desired weight in Tromsø 6 (2007–2008) minus desired weight in Tromsø 4 (1994–1995), with 95 % confidence intervals

^c^Discrepancy in difference (CI): Actual difference minus Difference in desired weight, with 95 % confidence intervals

**Table 5:** Longitudinal analyses of 13-year change in weight (kg), desired weight (kg) and difference between the two (kg), among women according to BMI category in Tromsø 4

	BMI <25				BMI 25-29.99				BMI ≥30			
	n	Actual difference^a^	Difference in desired weight^b^	Discrepancy in difference (CI)^c^	n	Actual difference^a^	Difference in desired weight^b^	Discrepancy in difference (CI)^c^	n	Actual difference^a^	Difference in desired weight^b^	Discrepancy in difference (CI)^c^
25–29	338	7.7	4.9	2.9 (2.3, 3.4)	97	9.1	6.2	2.9 (1.6, 4.4)	25	8.7	6.3	2.4 (-1.6, 6.0)
30–34	241	6.0	3.9	2.1 (1.4, 2.7)	76	7.6	5.1	2.5 (0.6, 4.0)	19	7.9	5.4	2.5 (-2.4, 7.6)
35–39	249	6.1	3.8	2.3 (1.8, 3.0)	85	6.3	5.3	1.0 (-0.5, 2.4)	21	10.2	6.2	4.0 (0.1, 7.6)
40–44	239	5.3	3.3	2.0 (1.4, 2.6)	106	6.0	4.3	1.7 (0.3, 3.2)	33	4.9	3.0	1.9 (-1.4, 4.7)
45–49	367	4.8	3.1	1.7 (1.2, 2.1)	239	5.3	3.7	1.6 (1.0, 2.3)	71	3.4	3.4	0.1 (-2.5, 2.2)
50–54	311	4.5	2.7	1.8 (1.3, 2.5)	228	4.5	3.5	1.1 (0.2, 2.0)	89	4.1	2.6	1.5 (-0.8, 4.0)
55–59	183	2.6	1.7	1.0 (0.4, 1.6)	159	2.7	2.7	0.0 (-1.2, 0.9)	57	−0.1	2.5	−2.6 (-5.1, -0.7)
60–64	114	0.9	0.4	0.5 (-0.5, 1.4)	128	2.8	1.7	1.0 (-0.1, 2.1)	38	0.6	1.8	−1.1 (-3.2, 1.5)
65–69	71	−0.6	0.1	−0.7 (-1.8, 0.6)	69	1.0	0.4	0.6 (-0.5, 2.1)	36	−2.1	1.1	−3.1 (-5.1, -1.0)
Total	2113	5.0	3.2	1.8 (1.6, 2.0)	1187	4.9	3.6	1.3 (0.9, 1.6)	389	3.3	3.1	0.2 (-0.7, 1.0)

^a^Actual difference: Measured body weight in Tromsø 6 (2007–2008) minus measured weight in Tromsø 4 (1994–1995), with 95 % confidence intervals

^b^Difference in desired weight: Desired weight in Tromsø 6 (2007–2008) minus desired weight in Tromsø 4 (1994–1995), with 95 % confidence intervals

^c^Discrepancy in difference (CI): Actual difference minus Difference in desired weight, with 95 % confidence intervals

For women (Table 5), we observed similar results as for men, but the difference between change in actual- and change in desired weight was significantly associated with age for all categories of BMI (*p* < 0.01). The difference between the actual and desired change in weight was highly significantly associated with category of BMI (*p* < 0.001) and, as for men, there was no significant interaction with BMI category regarding the relationship between age and the difference between the actual and desired change in weight (*p* = 0.2).

## Discussion

The results from this population-based longitudinal study suggest that increase in actual body weight is accompanied by an increase in desired bodyweight. The changes in desired body weight was largest for young people, with women consistently demonstrating a larger increase in desired body weight than men do. Furthermore, there was a quite consistent pattern of reduced difference between change in actual body weight and change in desired body weight with increasing age, suggesting that older age groups adapt more to weight gain than do younger age groups. Females seem in general to adapt more to increases in actual body weight than men do.

Our cross-sectional analyses of actual- and desired body weight in Tromsø 4 found that normal weight men of all age groups were quite satisfied with their bodyweight, while normal weight women were less so. This is in accordance with the findings of Blake et al.; more women than men in all BMI categories were found to be dissatisfied with their weight [9]. Similar differences in weight satisfaction between genders have been found in several studies [12, 13, 24, 25]. A suggested explanation has been the culturally different ideal body images between men and women, where the ideal female body shape is slim and lean, whereas for males it is lean and muscular [12]. This might also serve as an explanation of why the difference between actual- and desired body weight in the cross-sectional analyses was larger for women than for men across all categories of BMI. To some extent this discrepancy could suggest a higher potential for weight loss among women than men, but evidence is mixed as to whether difference between actual- and desired body weight is associated with greater motivation for weight loss [10, 26]. However, two American studies found that the association between obesity and intention for weight loss is stronger among women than men, with ethnic differences [27], and that more obese women than obese men have intentions to lose weight [28]. Since the Tromsø study is based on a largely homogenous group of Caucasians, ethnic differences were not explored, but for obese men and women the difference between actual- and desired bodyweight was higher among women (19.6 kg) than men (16.3 kg) suggesting dissatisfaction with bodyweight is larger among obese women than obese men. Accordingly, similar differences in intentions to lose weight may be expected, but intention to lose weight was not investigated in the present study. However, studies have found that lower satisfaction with weight, i.e. a large difference between actual- and desired bodyweight, and high weight loss expectations are associated with attrition from weight loss programs, particularly among the obese [29–32]. As such, the large differences between actual- and desired bodyweight observed among the obese should not be equalized to expected weight loss if obese individuals were included in a weight loss program. Rather this large discrepancy may be regarded as a long-term goal for the individual, or as a clear example of how obese people are not satisfied with being obese.

In our longitudinal analyses, we found that both desired body weight and actual body weight increased in all age-groups of both genders (except women aged 65–69) over a 13-year period (1994–1995 to 2007–2008) (Table 3). For both genders and all age groups except the oldest females, increase in actual body weight was higher than increase in desired body weight, and the difference between change in actual body weight and change in desired body weight was significantly associated with age. Similarly, Millstein et al. found that body dissatisfaction declined with age for women, but not for men [13]. It appears that with increasing age, both men and women adapt more to weight gain. Consequently, weight reduction interventions may yield higher results when targeting younger age groups, since older age groups seem less motivated for weight reduction.

While our cross-sectional results found obese men and women to be very dissatisfied with their body weight, at values similar to those found by Green et al. [33], our longitudinal analyses found that obese men and women experienced a smaller weight gain than those that were normal weight and overweight at baseline. The largest differences between changes in actual- and desired body weight were found among the normal- and overweight. This may be explained by these groups of people having had the largest increase in actual body weight.

Our study has confirmed cross-sectional and longitudinal trends in body weight and changes in body weight found in Norway [14, 18], and extended the knowledge to include changes in desired bodyweight. Previous cross-sectional studies conducted in Norway and Canada has investigated desired weight using a different approach, in which case the variable under analysis has been Desired Body Mass Index (DBMI), computed based on self-reported desired body weight and measured height [33, 34]. Also, an American study found secular increases in both actual- and desired body weight over a 9-year period, but this study relied on self-reported data on both height and weight [19].

We acknowledge some limitations in our study. Desired weight is highly subjective, but is hard to measure other than through questionnaire data or by interview. Other studies of weight satisfaction, using for instance a scale of satisfaction or visual body images, may capture other underlying factors not specific to bodyweight, such as social desirability [26]. Finally, although the subjects included in the longitudinal analyses did not differ much regarding desired weight from those who were not possible to include, we still have to acknowledge that the participants included in our study may not have been completely representative of the Tromsø 4- population aged 25–69.

However, the study also has several strengths. To our knowledge, this is the first study to investigate longitudinal changes in desired weight and adaptation to weight gain, and therefore represents new knowledge to the field of weight satisfaction. Furthermore, both surveys had high participation rates and height and weight were measured rather than self-reported; people often underestimate weight and overestimate height [35, 36]. We consider it more relevant to include in the analyses the actual weight the subject would be satisfied with rather than the BMI they would like to have, as BMI is an abstract measure for most people.

## Conclusion

In conclusion, we have found that in Tromsø 4 (1994–1995), BMI category is for both sexes a better predictor for difference between desired body weight and actual body weight than age, with weight satisfaction being higher among normal weight than overweight and lowest for obese. The longitudinal analyses of changes during 13 years from Tromsø 4 (1994–1995) to Tromsø 6 (2007–2008) found age group to be a better predictor than BMI category for the difference between change in body weight and change in desired bodyweight. Our findings suggest that older people adapt more to changes in weight than younger people, irrespective of BMI category. The introduction of desired body weight as a means to assess acceptance of weight gain in longitudinal analyses represents new opportunities for interventions specifically targeting those that seem to adapt most to weight gain, and further research on this topic is encouraged.

## Additional file

Additional file 1: Table S1. Cross-sectional analysis of measured and desired weight, and the difference between them, among men in Tromsø 5 (2001–2002). **Table S2.** Cross-sectional analysis of measured and desired weight, and the difference between them, among women in Tromsø 5 (2001–2002). **Table S3.** Cross-sectional analysis of measured and desired weight, and the difference between them, among men in Tromsø 6 (2007–2008). **Table S4.** Cross-sectional analysis of measured and desired weight, and the difference between them, among women in Tromsø 6 (2007–2008). (DOCX 27 kb)

## Abbreviation

BMI: Body mass index
