# Supplementary material for: Longitudinal changes in desired body weight compared to changes in body weight: evidence of adaptation to weight gain?
Source: BMC Obes. 2016 Sep 21;3:40. doi: 10.1186/s40608-016-0120-6 (PMC5031254; doi:10.1186/s40608-016-0120-6)
Supplement: Additional file 1: Table S1. — Cross-sectional analysis of measured and desired weight, and the difference between them, among men in Tromsø 5 (2001–2002). Table S2. Cross-sectional analysis of measured and desired weight, and the difference between them, among women in Tromsø 5 (2001–2002). Table S3. Cross-sectional analysis of measured and desired weight, and the difference between them, among men in Tromsø 6 (2007–2008). Table S4. Cross-sectional analysis of measured and desired weight, and the difference between them, among women in Tromsø 6 (2007–2008). (DOCX 27 kb) [file 40608_2016_120_MOESM1_ESM.docx]

Additional tables to the submitted manuscript:

Longitudinal Changes in Desired Body Weight Compared to Changes in Body Weight: Evidence of Adaptation to Weight Gain?

Nils Abel Aars*^1^, Bjarne K. Jacobsen^1^

Address: ^1^Department of Community Medicine, UiT – The Arctic University of Norway, Tromsø, Norway.

Contact:

Nils Abel Aars* - nils.a.aars@uit.no

Phone: (+47) 412 39 138

Bjarne K. Jacobsen – bjarne.jacobsen@uit.no

* Corresponding author

| Appendix Table 1. Cross-sectional analysis of measured and desired weight, and the difference between them, among men in Tromsø 5 (2001-2002).* | | | | | | | | | | | | | | | | |
| --- | --- | --- | --- | --- | --- | --- | --- | --- | --- | --- | --- | --- | --- | --- | --- | --- |
|  | Total | | | | BMI < 25 | | | | BMI 25-29.99 | | | | BMI ≥ 30 | | | |
| Age group | *n* | Weight | Desired weight | Difference (CI)^#^ | *n* | Weight | Desired weight | Difference (CI)^#^ | *n* | Weight | Desired weight | Difference (CI)^#^ | *n* | Weight | Desired weight | Difference (CI)^#^ |
| 30-34 | 176 | 84.5 | 80.4 | 4.1 (3.3, 5.5) | 75 | 75.0 | 76.2 | -1.2 (-2.1, -0.3) | 68 | 86.0 | 80.6 | 5.4 (4.5, 6.4) | 33 | 102.9 | 89.3 | 13.6 (11.3, 15.7) |
| 35-39 | 37 | 82.0 | 77.0 | 5.0 (2.6, 7.1) | 12 | 74.5 | 74.6 | -0.1 (-2.1, 1.6) | 22 | 82.7 | 76.3 | 6.4 (4.5, 8.4) | 3 | 106.5 | 91.7 | 14.8 (11.6, 17.0) |
| 40-44 | 248 | 84.9 | 79.2 | 5.7 (4.8, 6.7) | 85 | 75.7 | 75.3 | 0.4 (-0.3, 1.4) | 125 | 86.0 | 80.0 | 5.9 (5.3, 6.7) | 38 | 102.3 | 85.5 | 16.9 (14.4, 19.7) |
| 45-49 | 240 | 84.8 | 78.6 | 6.2 (5.2, 7.0) | 74 | 74.0 | 73.1 | 1.0 (0.2, 1.8) | 128 | 85.9 | 79.8 | 6.1 (5.4, 7.0) | 38 | 101.8 | 85.2 | 16.7 (14.3, 19.3) |
| 50-54 | 126 | 87.7 | 80.2 | 7.5 (6.2, 8.9) | 27 | 71.9 | 72.0 | -0.1 (-1.2, 1.1) | 67 | 86.0 | 79.8 | 6.2 (5.4, 7.2) | 32 | 104.7 | 88.1 | 16.6 (13.4, 20.2) |
| 55-59 | 201 | 87.6 | 80.7 | 6.8 (5.9, 7.8) | 36 | 72.5 | 72.0 | 0.5 (-0.4, 1.5) | 116 | 87.1 | 81.2 | 5.9 (5.3, 6.6) | 49 | 99.9 | 86.0 | 13.8 (11.4, 16.5) |
| 60-64 | 575 | 84.5 | 78.5 | 6.0 (5.5, 6.6) | 136 | 71.7 | 71.7 | 0.1 (-0.6, 0.6) | 313 | 84.3 | 79.0 | 5.3 (4.8, 5.5) | 126 | 98.6 | 84.4 | 14.2 (13.0, 15.4) |
| 65-69 | 500 | 81.5 | 77.0 | 4.4 (3.8, 5.0) | 154 | 70.2 | 70.9 | -0.7 (-1.2, -0.3) | 263 | 82.6 | 77.9 | 4.7 (4.2, 5.1) | 83 | 98.7 | 85.7 | 13.0 (11.6, 14.2) |
| 70-74 | 441 | 79.3 | 75.5 | 3.8 (3.3, 4.5) | 146 | 68.7 | 70.0 | -1.3 (-2.0, -0.5) | 228 | 81.2 | 76.8 | 4.4 (4.0, 5.1) | 67 | 95.7 | 82.7 | 13.0 (11.4, 14.7) |
| 75-79 | 303 | 77.3 | 74.3 | 3.0 (2.4, 3.8) | 123 | 68.1 | 69.7 | -1.6 (-2.3, -0.6) | 137 | 80.3 | 75.9 | 4.4 (3.4, 5.3) | 43 | 94.2 | 82.2 | 12.0 (9.9, 14.1) |
| 80-86 | 93 | 79.5 | 76.5 | 3.0 (0.8, 4.8) | 35 | 66.6 | 69.9 | -3.3 (-5.2, -1.6) | 42 | 84.0 | 79.8 | 4.2 (3.0, 5.8) | 16 | 95.8 | 82.3 | 13.6 (10.5, 16.8) |
| Total | 2940 | 82.7 | 77.7 | 5.0 (4.7, 5.3) | 903 | 71.2 | 71.8 | -0.6 (-0.9, -0.4) | 1509 | 83.8 | 78.6 | 5.2 (5.0, 5.4) | 528 | 99.1 | 85.0 | 14.1 (13.5, 14.7) |

*: Results for all 2940 men and according to three categories of BMI (normal weight, overweight and obese).

#Difference (CI): Difference between actual, measured weight and self-reported desired weight, with 95% confidence intervals.

| Appendix Table 2. Cross-sectional analysis of measured and desired weight, and the difference between them, among women in Tromsø 5 (2001-2002).* | | | | | | | | | | | | | | | | |
| --- | --- | --- | --- | --- | --- | --- | --- | --- | --- | --- | --- | --- | --- | --- | --- | --- |
|  | Total | | | | BMI < 25 | | | | BMI 25-29.99 | | | | BMI ≥ 30 | | | |
| Age group | *n* | Weight | Desired weight | Difference (CI)^#^ | *n* | Weight | Desired weight | Difference (CI)^#^ | *n* | Weight | Desired weight | Difference (CI)^#^ | *n* | Weight | Desired weight | Difference (CI)^#^ |
| 30-34 | 272 | 67.9 | 61.4 | 6.5 (5.5, 7.6) | 169 | 61.5 | 59.0 | 2.5 (2.1, 3.0) | 71 | 72.0 | 63.0 | 9.1 (8.2, 10.2) | 32 | 91.9 | 70.3 | 21.5 (17.8, 25.1) |
| 35-39 | 67 | 69.5 | 62.4 | 7.1 (5.4, 8.7) | 39 | 62.6 | 58.6 | 4.0 (2.5, 5.1) | 20 | 73.5 | 64.8 | 8.7 (7.3, 10.9) | 8 | 93.5 | 75.0 | 18.5 (11.4, 25.7) |
| 40-44 | 309 | 69.1 | 62.1 | 7.0 (6.1, 8.0) | 174 | 61.4 | 58.7 | 2.7 (2.2, 3.2) | 99 | 73.2 | 64.3 | 8.9 (8.1, 9.8) | 36 | 94.5 | 72.5 | 22.0 (19.4, 26.0) |
| 45-49 | 299 | 68.5 | 62.0 | 6.5 (5.8, 7.5) | 157 | 61.0 | 58.4 | 2.7 (2.2, 3.2) | 112 | 73.0 | 64.7 | 8.3 (7.7, 9.1) | 30 | 91.2 | 71.0 | 20.2 (16.4, 22.9) |
| 50-54 | 88 | 69.5 | 62.3 | 7.1 (5.9, 8.8) | 47 | 61.8 | 58.6 | 3.2 (2.2, 4.0) | 30 | 73.7 | 64.1 | 9.6 (8.1, 11.2) | 11 | 90.9 | 73.4 | 17.5 (14.1, 21.2) |
| 55-59 | 559 | 72.5 | 63.7 | 8.9 (8.2, 9.6) | 197 | 60.7 | 58.7 | 2.0 (1.6, 2.5) | 228 | 73.0 | 64.7 | 8.3 (7.8, 9.0) | 134 | 89.1 | 69.3 | 19.9 (18.5, 21.9) |
| 60-64 | 703 | 70.2 | 62.9 | 7.3 (6.6, 8.1) | 276 | 60.0 | 58.5 | 1.4 (1.0, 1.8) | 272 | 71.1 | 64.2 | 6.9 (6.4, 7.4) | 155 | 86.9 | 68.5 | 18.4 (16.8, 20.2) |
| 65-69 | 542 | 69.3 | 62.9 | 6.4 (5.8, 7.0) | 188 | 58,4 | 58.0 | 0.4 (-0.3, 0.8) | 237 | 70.7 | 64.1 | 6.6 (6.0, 7.0) | 117 | 84.2 | 68.4 | 15.8 (14.2, 17.1) |
| 70-74 | 473 | 68.8 | 63.3 | 5.6 (4.8, 6.3) | 173 | 57.7 | 57.8 | -0.2 (-0.8, 0.5) | 182 | 69.4 | 63.8 | 5.6 (5.1, 6.1) | 118 | 84.4 | 70.5 | 13.9 (12.8, 15.1) |
| 75-79 | 346 | 68.8 | 64.0 | 4.8 (3.8, 5.5) | 112 | 57.0 | 57.8 | -0.9 (-1.7, -0.3) | 143 | 69.4 | 64.9 | 4.5 (3.9, 5.1) | 91 | 82.4 | 70.4 | 12.0 (10.8, 13.7) |
| 80-88 | 92 | 68.1 | 64.0 | 4.1 (2.7, 5.4) | 24 | 56.0 | 57.5 | -1.5 (-3.1, -0.2) | 41 | 67.9 | 64.0 | 3.9 (2.8, 5.0) | 27 | 79.1 | 69.7 | 9.4 (6.5, 12.7) |
| Total | 3750 | 69.6 | 62.9 | 6.7 (6.5, 7.0) | 1556 | 59.9 | 58.4 | 1.5 (1.3, 1.7) | 1435 | 71.3 | 64.3 | 7.0 (6.8, 7.3) | 759 | 86.5 | 69.7 | 16.8 (16.2, 17.6) |

*: Results for all 3750 women and according to three categories of BMI (normal weight, overweight and obese).

#Difference (CI): Difference between actual, measured weight and self-reported desired weight, with 95% confidence intervals.

| Appendix Table 3. Cross-sectional analysis of measured and desired weight, and the difference between them, among men in Tromsø 6 (2007-2008).* | | | | | | | | | | | | | | | | |
| --- | --- | --- | --- | --- | --- | --- | --- | --- | --- | --- | --- | --- | --- | --- | --- | --- |
|  | Total | | | | BMI < 25 | | | | BMI 25-29.99 | | | | BMI ≥ 30 | | | |
| Age group | *n* | Weight | Desired weight | Difference (CI)^#^ | *n* | Weight | Desired weight | Difference (CI)^#^ | *n* | Weight | Desired weight | Difference (CI)^#^ | *n* | Weight | Desired weight | Difference (CI)^#^ |
| 30-34 | 77 | 88.6 | 81.2 | 7.5 (5.7, 9.1) | 27 | 77.6 | 76.8 | 0.8 (-0.4, 2.0) | 35 | 90.3 | 81.2 | 9.1 (7.6, 10.4) | 15 | 104.5 | 88.9 | 15.7 (13.1, 19.3) |
| 35-39 | 104 | 90.8 | 82.5 | 8.3 (6.8, 9.6) | 23 | 76.3 | 75.2 | 1.2 (-0.2, 2.8) | 53 | 88.5 | 81.8 | 6.7 (5.7, 7.8) | 28 | 107.1 | 89.9 | 17.1 (13.9, 20.2) |
| 40-44 | 915 | 87.7 | 80.7 | 7.0 (6.4, 7.6) | 275 | 74.7 | 74.5 | 0.2 (-0.3, 0.7) | 440 | 87.8 | 81.2 | 6.6 (6.2, 7.2) | 200 | 105.4 | 88.1 | 17.3 (16.4, 18.4) |
| 45-49 | 499 | 88.2 | 80.8 | 7.4 (6.5, 8.2) | 125 | 75.3 | 74.4 | 1.0 (0.2, 1.5) | 282 | 87.6 | 80.1 | 6.9 (6.5, 7.4) | 92 | 107.5 | 89.6 | 17.8 (15.6, 20.4) |
| 50-54 | 447 | 87.1 | 79.9 | 7.2 (6.3, 8.0) | 114 | 74.8 | 74.5 | 0.3 (-0.5, 1.1) | 234 | 86.4 | 80.2 | 6.2 (5.7, 6.6) | 99 | 103.2 | 85.6 | 17.7 (16.0, 19.0) |
| 55-59 | 505 | 87.1 | 79.8 | 7.3 (6.5, 8.1) | 116 | 72.9 | 72.1 | 0.9 (0.1, 1.8) | 273 | 86.1 | 79.7 | 6.4 (5.9, 7.0) | 116 | 103.6 | 88.0 | 15.6 (14.0, 17.4) |
| 60-64 | 985 | 86.9 | 79.5 | 7.4 (7.0, 7.9) | 218 | 72.3 | 71.9 | 0.4 (-0.1, 0.8) | 518 | 86.0 | 79.8 | 6.2 (5.8, 6.5) | 249 | 101.5 | 85.4 | 16.2 (15.2, 17.0) |
| 65-69 | 693 | 85.2 | 78.6 | 6.6 (6.0, 7.1) | 163 | 71.3 | 71.5 | -0.2 (-0.8, 0.3) | 367 | 84.9 | 79.1 | 5.8 (5.4, 6.3) | 163 | 99.7 | 84.6 | 15.0 (14.0, 16.1) |
| 70-74 | 411 | 82.7 | 77.4 | 5.3 (4.6, 6.0) | 114 | 70.1 | 71.4 | -1.3 (-1.8, -0.6) | 214 | 83.5 | 78.4 | 5.1 (4.6, 5.6) | 83 | 97.9 | 83.3 | 14.7 (13.2, 16.5) |
| 75-79 | 245 | 79.7 | 75.1 | 4.6 (3.6, 5.6) | 90 | 69.7 | 70.4 | -0.7 (-1.3, -0.1) | 118 | 82.1 | 76.7 | 5.4 (4.7, 5.9) | 37 | 96.8 | 81.7 | 15.1 (12.5, 16.7) |
| 80-87 | 131 | 78.7 | 74.7 | 4.0 (2.7, 5.1) | 46 | 69.2 | 70.8 | -1.6 (-2.7, -0.4) | 68 | 81.1 | 75.7 | 5.5 (4.4, 6.2) | 17 | 94.6 | 81.5 | 13.1 (11.2, 15.2) |
| Total | 5012 | 86.2 | 79.4 | 6.8 (6.7, 7.1) | 1311 | 72.9 | 72.8 | 0.1 (-0.1, 0.3) | 2602 | 86.0 | 79.8 | 6.2 (6.0, 6.4) | 1099 | 102.5 | 86.2 | 16.2 (15.7, 16.7) |

*: Results for all 5012 men and according to three categories of BMI (normal weight, overweight and obese).

#Difference (CI): Difference between actual, measured weight and self-reported desired weight, with 95% confidence intervals.

| Appendix Table 4. Cross-sectional analysis of measured and desired weight, and the difference between them, among women in Tromsø 6 (2007-2008).* | | | | | | | | | | | | | | | | |
| --- | --- | --- | --- | --- | --- | --- | --- | --- | --- | --- | --- | --- | --- | --- | --- | --- |
|  | Total | | | | BMI < 25 | | | | BMI 25-29.99 | | | | BMI ≥ 30 | | | |
| Age group | *n* | Weight | Desired weight | Difference (CI)^#^ | *n* | Weight | Desired weight | Difference (CI)^#^ | *n* | Weight | Desired weight | Difference (CI)^#^ | *n* | Weight | Desired weight | Difference (CI)^#^ |
| 30-34 | 104 | 69.6 | 62.6 | 7.0 (5.6, 8.5) | 59 | 61.0 | 58.3 | 2.8 (1.8, 3.5) | 29 | 74.7 | 65.2 | 9.5 (8.4, 10.7) | 16 | 92.0 | 73.9 | 18.1 (14.7, 22.7) |
| 35-39 | 140 | 71.8 | 63.2 | 8.6 (7.0, 10.3) | 66 | 62.5 | 59.2 | 3.4 (2.7, 4.0) | 47 | 75.5 | 66.9 | 8.6 (7.5, 9.4) | 27 | 88.0 | 66.7 | 21.3 (16.5, 25.9) |
| 40-44 | 1113 | 71.8 | 63.4 | 8.4 (7.9, 8.9) | 514 | 61.8 | 59.0 | 2.8 (2.6, 3.2) | 397 | 73.9 | 64.8 | 9.1 (8.7, 9.4) | 202 | 93.3 | 72.1 | 21.2 (19.7, 22.7) |
| 45-49 | 522 | 73.0 | 64.0 | 9.0 (8.3, 9.8) | 223 | 62.2 | 59.2 | 3.0 (2.6, 3.3) | 185 | 73.8 | 64.9 | 8.9 (8.2, 9.6) | 114 | 92.6 | 71.8 | 20.8 (18.9, 22.9) |
| 50-54 | 554 | 70.8 | 63.4 | 7.4 (6.7, 8.1) | 243 | 61.7 | 59.1 | 2.7 (2.3, 3.0) | 229 | 72.6 | 64.7 | 7.9 (7.5, 8.3) | 82 | 92.5 | 72.4 | 20.1 (17.8, 21.5) |
| 55-59 | 517 | 71.5 | 63.7 | 7.8 (7.1, 8.5) | 223 | 61.6 | 58.9 | 2.7 (2.3, 3.1) | 195 | 73.7 | 65.5 | 8.2 (7.6, 8.7) | 99 | 89.7 | 70.9 | 18.8 (17.3, 20.8) |
| 60-64 | 1044 | 73.2 | 64.5 | 8.7 (8.2, 9.2) | 325 | 61.7 | 59.7 | 2.0 (1.6, 2.4) | 468 | 72.5 | 64.7 | 7.8 (7.5, 8.2) | 251 | 89.4 | 70.5 | 18.9 (17.7, 20.2) |
| 65-69 | 625 | 71.7 | 64.4 | 7.3 (6.4, 8.1) | 217 | 59.9 | 59.3 | 0.6 (-0.4, 1.4) | 262 | 72.3 | 65.2 | 7.1 (5.9, 7.9) | 146 | 88.2 | 70.4 | 17.7 (16.2, 19.2) |
| 70-74 | 408 | 70.0 | 63.5 | 6.8 (6.1, 7.5) | 135 | 58.7 | 58.2 | 0.5 (-0.2, 1.0) | 172 | 71.1 | 64.4 | 6.7 (6.1, 7.2) | 101 | 84.8 | 69.3 | 15.5 (14.2, 16.8) |
| 75-79 | 309 | 68.7 | 62.8 | 6.0 (5.0, 6.6) | 101 | 56.1 | 56.4 | -0.4 (-1.2, 0.5) | 127 | 70.0 | 64.1 | 5.9 (4.9, 6.4) | 81 | 82.6 | 68.6 | 14.0 (12.7, 15.7) |
| 80-87 | 202 | 67.6 | 63.1 | 4.5 (3.6, 5.9) | 67 | 55.8 | 57.2 | -1.4 (-2.5, -0.4) | 83 | 68.2 | 63.6 | 4.6 (3.8, 5.7) | 52 | 81.8 | 69.9 | 11.8 (10.0, 14.1) |
| Total | 5538 | 71.6 | 63.8 | 7.8 (7.6, 8.0) | 2173 | 61.0 | 58.9 | 2.1 (1.9, 2.3) | 2194 | 72.6 | 64.8 | 7.8 (7.6, 8.0) | 1171 | 89.3 | 70.8 | 18.5 (18.0, 19.1) |

*: Results for all 5538 women and according to three categories of BMI (normal weight, overweight and obese).

#Difference (CI): Difference between actual, measured weight and self-reported desired weight, with 95% confidence intervals.
